# Supplementary figures and images for: The clinical course of hospitalized moderately ill COVID-19 patients is mirrored by routine hematologic tests and influenced by renal transplantation
Source: PLoS One. 2021 Nov 18;16(11):e0258987. doi: 10.1371/journal.pone.0258987 (PMC8601535; doi:10.1371/journal.pone.0258987)

**Supplementary Figure 1 – Demographic data of the COVID-19 cohort.**


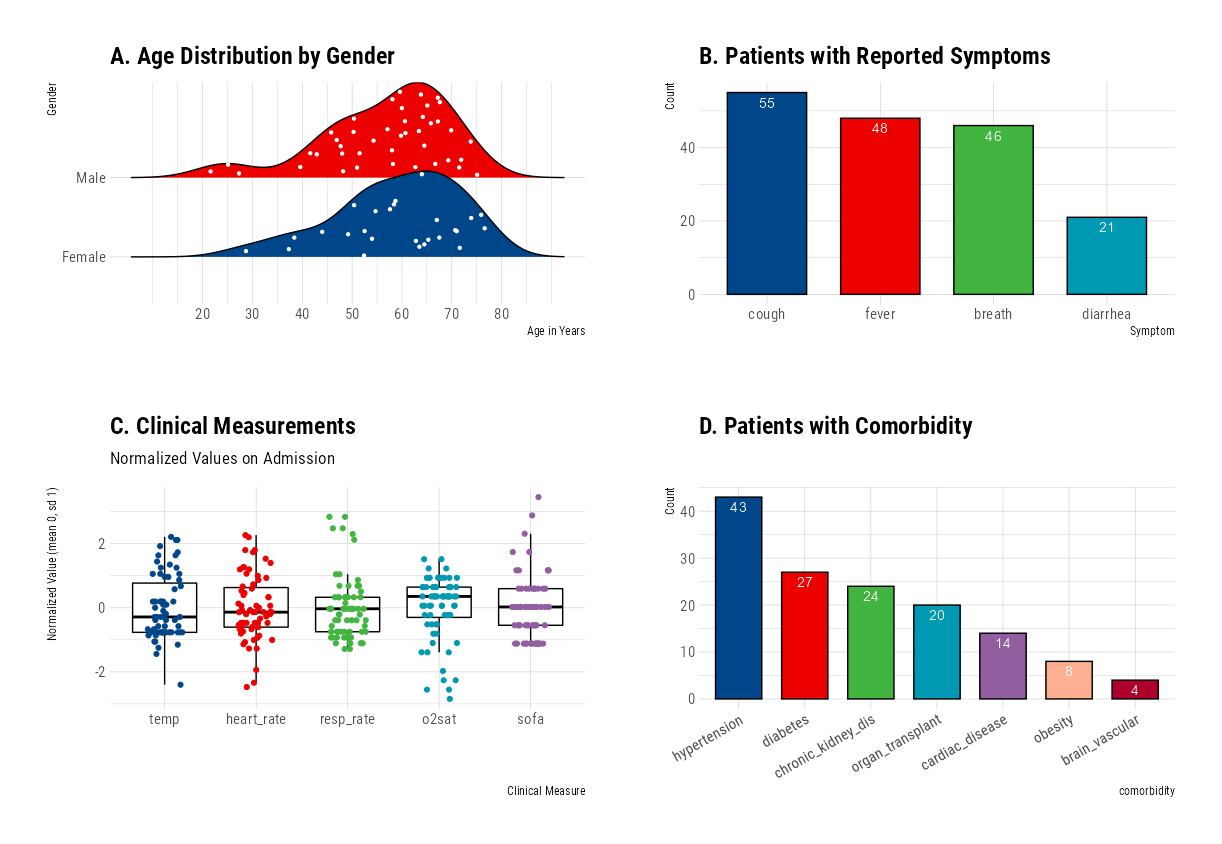

Supplement: S1 Fig — (DOCX) [file pone.0258987.s001.docx]
