# Supplementary material for: The clinical course of hospitalized moderately ill COVID-19 patients is mirrored by routine hematologic tests and influenced by renal transplantation
Source: PLoS One. 2021 Nov 18;16(11):e0258987. doi: 10.1371/journal.pone.0258987 (PMC8601535; doi:10.1371/journal.pone.0258987)

**Supplementary Figure 2 – A principal components model showing the separation in values for each of the laboratory tests between day 1 and day 3.**


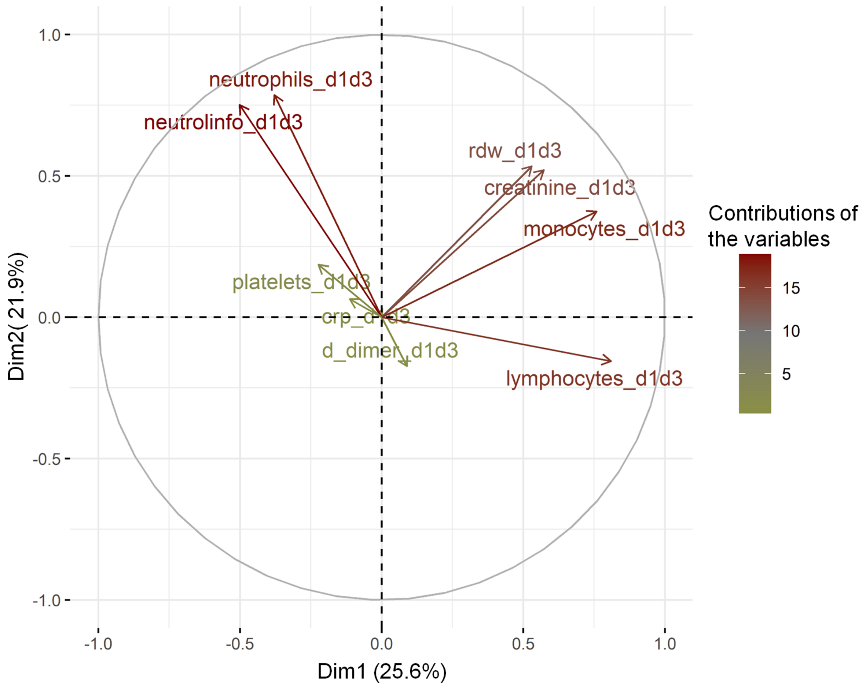

Supplement: S2 Fig — (DOCX) [file pone.0258987.s002.docx]
