# Supplementary material for: The clinical course of hospitalized moderately ill COVID-19 patients is mirrored by routine hematologic tests and influenced by renal transplantation
Source: PLoS One. 2021 Nov 18;16(11):e0258987. doi: 10.1371/journal.pone.0258987 (PMC8601535; doi:10.1371/journal.pone.0258987)

**Supplementary Figure 3 – CRP (A) and Creatinine (B) levels in COVID-19 patients accordingly to the clinical course and outcomes.**


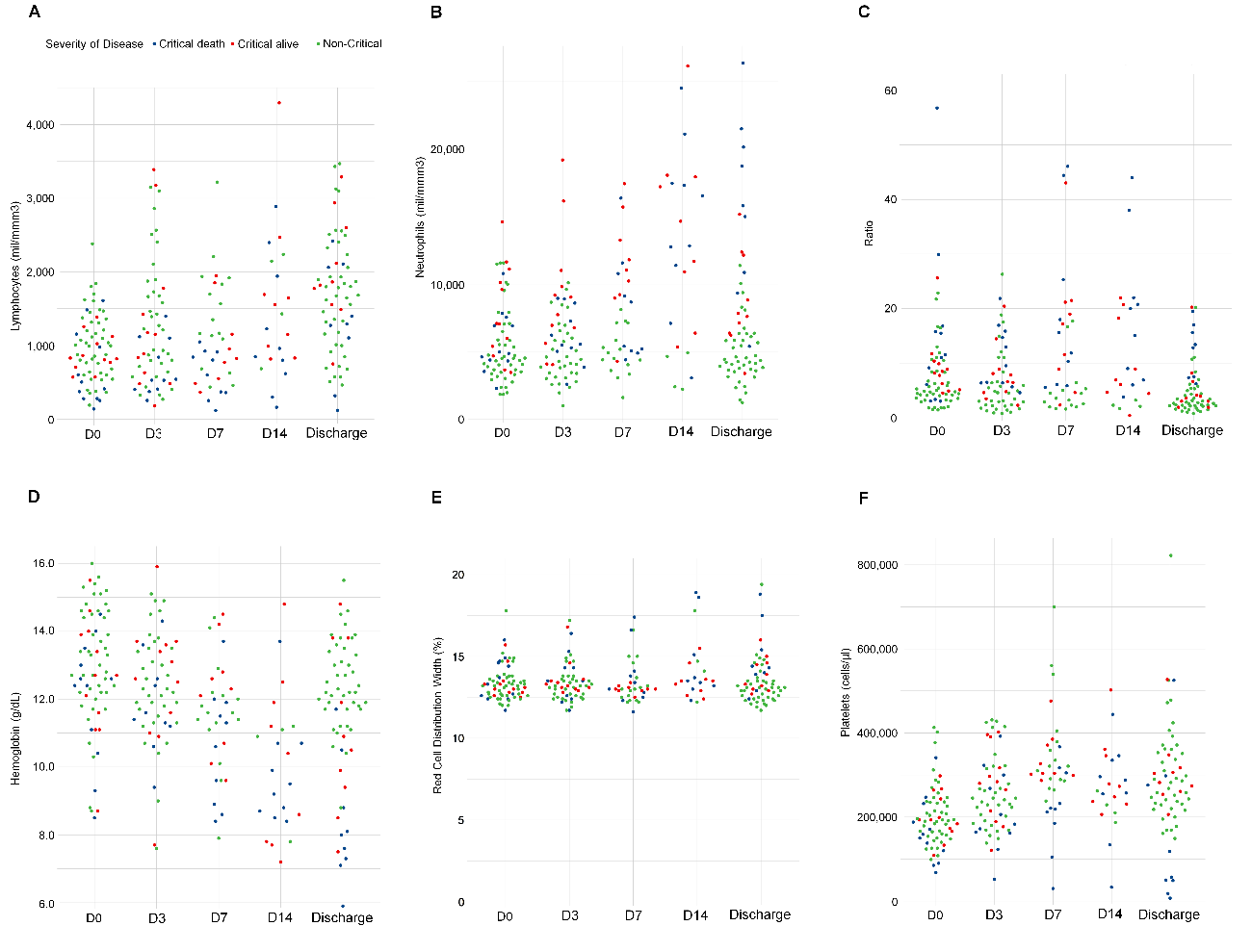

Supplement: S3 Fig — CRP (A) and Creatinine (B) levels in COVID-19 patients accordingly to the clinical course and outcomes. (DOCX) [file pone.0258987.s003.docx]
