# Supplementary material for: The clinical course of hospitalized moderately ill COVID-19 patients is mirrored by routine hematologic tests and influenced by renal transplantation
Source: PLoS One. 2021 Nov 18;16(11):e0258987. doi: 10.1371/journal.pone.0258987 (PMC8601535; doi:10.1371/journal.pone.0258987)
Supplement: S1 Table — (DOCX) [file pone.0258987.s004.docx]

| **Supplementary table 1.** Routine laboratory tests of the cohort according to the clinical course and outcomes of the disease | | | | | |
| --- | --- | --- | --- | --- | --- |
|  | **Critical (N = 24)** | | **Non-critical (N = 44)** | **P-Value *** | **P-Value **** |
|  | **Hospital discharge (N = 12)** | **Hospital death (N = 12)** |  |  |  |
| **Laboratory Admission** | | | | | |
| Lymphocytes, cells/µl, Mean (SD) | 893 (255) | 671 (507) | 1,018 (471.62) | 0,194 | **0,043** |
| Neutrophils, cells/µl | 7,856 (3,528) | 5,735 (2,324) | 5,175 (2,682.33) | 0,096 | **0,019** |
| Monocytes, cells/µl | 424 (194) | 376 (273) | 381 (268.99) | 0,624 | 0,513 |
| Neutrophil-Lymphocyte Ratio | 9.2 (5.6) | 15.1 (15.1) | 6.5 (5.0) | 0,544 | **0,003** |
| Platelets, cells/µl | 201,833 (56,413) | 165,750 (78158) | 207,750 (71,257) | 0,208 | 0,230 |
| Hemoglobin, g/dL | 12.6 (1.8) | 12.0 (1.8) | 13.1 (1.6) | 0,441 | 0,073 |
| Hematocrit, (%) | 37.7 (5.8) | 36.6 (4.8) | 39.4 (4.8) | 0,613 | 0,128 |
| Red Cell Distribution Width, (%) | 13.3 (0.9) | 13.6 (1.3) | 13.3 (1.0) | 0,582 | 0,694 |
| Creatinine, mg/dL | 1.5 (1.6) | 2.2 (1.6) | 1.13 (0.6) | 0,184 | 0,311 |
| C-Reactive Protein, mg/L | 132.3 (98.7) | 141.2 (98.1) | 106.1 (75.9) | 0,834 | 0,090 |
| Lactate, mg/dL | 18.6 (6.1) | 15.9 (9.9) | 12.2 (6.0) | 0,556 | 0,060 |
| D-dimer, µg/mL FEU | 2.5 (2.6) | 2.0 (2.1) | 1.8 (2.1) | 0,909 | 0,792 |
| **Laboratory D3** | | | | | |
| Lymphocytes, cells/µl, Mean (SD) | 1,298 (1,026) | 680 (374) | 1,352 (821) | 0,085 | 0,053 |
| Neutrophils, cells/µl | 9,123 (4,585) | 6,143 (2,085) | 4,853 (2,250) | 0,049 | **0,001** |
| Monocytes, cells/µl | 904 (1010) | 312 (171) | 419 (182) | **0,008** | 0,918 |
| Neutrophil-Lymphocyte Ratio | 16.2 (29.0) | 11.0 (5.7) | 5.9 (5.8) | 0,460 | **0,001** |
| Platelets, cells/µl | 277,833 (90,904) | 213,181 (98,177) | 257,485 (84,800) | 0,116 | 0,745 |
| Hemoglobin, g/dL | 12.4 (2.0) | 11.8 (1.4) | 12.4 (1.6) | 0,417 | 0,055 |
| Hematocrit, (%) | 38.0 (6.4) | 35.9 (4.0) | 37.2 (4.8) | 0,379 | 0,063 |
| Red Cell Distribution Width, (%) | 13.6 (1.1) | 13.6 (1.4) | 13.2 (1.0) | 0,947 | 0,375 |
| Creatinine, mg/dL | 2.2 (2.7) | 2.3 (1.6) | 1.0 (0.4) | 0,942 | **0,033** |
| C-Reactive Protein, mg/L | 105.9 (79.9) | 162.4 (137.4) | 64.4 (49.5) | 0,352 | 0,052 |
| Lactate, mg/dL | 17.8 (6.4) | 17.0 (1.0) | 16.5 (7.3) | 0,842 | 0,066 |
| D-dimer, µg/mL FEU | 1.6 (0.7) | 1.3 (0.7) | 2.9 (4.0) | 0,462 | 0,801 |
| **Laboratory D7** | | | | | |
| Lymphocytes, cells/µl, Mean (SD) | 988 (570) | 622 (326) | 1,405 (731) | 0,191 | **0,007** |
| Neutrophils, cells/µl | 11,321 (3,900) | 8,137 (3,918) | 4,974 (1,767) | 0,094 | **0,0004** |
| Monocytes, cells/µl | 849 (366) | 402.4 (202.8) | 537 (258) | **0,004** | 0,77 |
| Neutrophil-Lymphocyte Ratio | 16.5 (12.1) | 18.90 (15.2) | 5.1 (4.9) | 0,806 | **0,0001** |
| Platelets, cells/µl | 339,444 (61,414) | 219,300 (99,441) | 366,562 (127,370) | **0,006** | 0,069 |
| Hemoglobin, g/dL | 12.1 (1.7) | 10.6 (1.7) | 11.5 (1.5) | 0,084 | 0,055 |
| Hematocrit, (%) | 36.7 (5.0) | 33.0 (5.6) | 35.2 (4.3) | 0,152 | 0,074 |
| Red Cell Distribution Width, (%) | 13.0 (0.2) | 13.7 (1.8) | 13.3 (1.2) | 0,250 | 0,778 |
| Creatinine, mg/dL | 1.6 (0.8) | 2.6 (1.4) | 1.0 (0.4) | 0,071 | **0,016** |
| C-Reactive Protein, mg/L | 56.2 (33.9) | 165.3 (138.3) | 53.3 (47.6) | 0,186 | 0,073 |
| Lactate, mg/dL | 18.3 (4.0) | 16.5 (6.3) | 395,0 | *NA* | *NA* |
| **Hospital Discharge** | | | | | |
| Lymphocytes, cells/µl, Mean (SD) | 2,018 (748) | 1,343 (780) | 1,692 (800) | 0,071 | 0,977 |
| Neutrophils, cells/µl | 8,696 (3,523) | 15,905 (6,590) | 4,862 (2,194) | **0,008** | **<0.0001** |
| Monocytes, cells/µl | 788 (227) | 1,138 (1,022) | 584 (218) | **0,018** | **0,01** |
| Neutrophil-Lymphocyte Ratio | 5.6 (5.5) | 21.1 (26.6) | 3.8 (3.5) | **0,007** | **0,0003** |
| Platelets, cells/µl | 307,900 (86,295) | 155,333 (17,5319) | 302,023 (122,233) | **0,037** | 0,24 |
| Hemoglobin, g/dL | 11.1 (2.4) | 8.3 (1.7) | 12.46 (1.2) | **0,012** | **<0.0001** |
| Hematocrit, (%) | 33.6 (7.1) | 25.8 (6.5) | 37.61 (3.7) | **0,024** | **0,0004** |
| Red Cell Distribution Width, (%) | 13.9 (1.0) | 14.8 (2.0) | 13.21 (1.2) | 0,462 | **0,003** |
| Creatinine, mg/dL | 1.2 (0.5) | 3.3 (0.8) | 1.02 (0.3) | **0,000** | **0,007** |
| C-Reactive Protein, mg/L | 50.1 (38.6) | 70.6 (53.1) | 26.97 (22.3) | 0,455 | **0,001** |
|  |  |  |  |  |  |
| Mann-Whitney, t-test or chi-square were applied to determine the P value when comparing groups | | | |  |  |
| * Hospital discharge (N = 12) x Hospital death (N = 2) | |  |  |  |  |
| ** Critical (N = 24) x Non-critical (N = 44) | |  |  |  |  |
| SD = Standard Deviation | | | | | |
