# Supplementary material for: The clinical course of hospitalized moderately ill COVID-19 patients is mirrored by routine hematologic tests and influenced by renal transplantation
Source: PLoS One. 2021 Nov 18;16(11):e0258987. doi: 10.1371/journal.pone.0258987 (PMC8601535; doi:10.1371/journal.pone.0258987)
Supplement: S2 Table — (DOCX) [file pone.0258987.s005.docx]

| **Supplementary table 2**. Demographic, clinical and laboratory results of non-KT recipients COVID-19 patients accordingly to the clinical course of the disease | | | |
| --- | --- | --- | --- |
|  | **Critical (N = 14)** | **Non-critical (N = 34)** | **P-Value **** |
| **Demography** | | | |
| Male sex, n (ratio) | 9 (0.64) | 21 (0.61) | 0,870 |
| Age, Mean (SD) | 64.92 (7.84) | 55.97 (14.17) | **0,034** |
| **Admission data** | | | |
| Day of symptoms | 5.69 (2.62) | 8.09 (3.79) | **0,045** |
| Fever, n (ratio) | 8 (0.57) | 24 (0.70) | 0,369 |
| Cough | 10 (0.71) | 26 (0.76) | 0,714 |
| Shortness of breath | 11 (0.78) | 20 (0.58) | 0,194 |
| Diarrhea | 5 (0.35) | 9 (0.26) | 0,522 |
| Temperature, Mean (SD) | 36.7 (1.4) | 36.8 (0.9) | 0,432 |
| Cardiac Rate | 86.9 (15.5) | 88.2 (16.0) | 0,802 |
| Respiratory rate | 23.1 (5.3) | 24.9 (6.6) | 0,45 |
| SpO2 | 92.2 (3.6) | 92.8 (3.6) | 0,614 |
| Body mass index | 26.0 (4.5) | 30.4 (6.9) | **0,030** |
| SOFA score | 2.2 (2.2) | 1.3 (1.2) | 0,223 |
| **Comorbidities** | | | |
| Cardiac disease, n (ratio) | 3 (0.21) | 9 (0.26) | 0,714 |
| Chronic pulmonary disease | 3 (0.21) | 6 (0.17) | 0,76 |
| Diabetes | 7 (0.50) | 13 (0.38) | 0,452 |
| Chronic kidney disease | 3 (0.21) | 1 (0.02) | **0,035** |
| Hypertension | 10 (0.71) | 17 (0.50) | 0,174 |
| Obesity | 1 (0.07) | 6 (0.17) | 0,349 |
| Stroke | 1 (0.07) | 2 (0.05) | 0,870 |
| Charlson Comorbidity index, Mean (SD) | 4.5 (2.2) | 2.5 (2.1) | **0,009** |
| Hospital days | 31.2 (25.0) | 7.8 (5.7) | **0,043** |
| **Laboratory Admission** | | | |
| Lymphocytes, Mean (SD) | 845 (338) | 1,101 (458) | 0,066 |
| Neutrophils | 7,744 (3,544) | 5,182 (2,641) | **0,015** |
| Monocytes | 444 (234) | 355 (223) | 0,129 |
| Neutrophils/Lymphocytes | 10.4 (5.7) | 5.8 (4.7) | **0,002** |
| Platelets | 185,500 (68,971) | 205,588 (76,139) | 0,691 |
| Hemoglobin | 12.3 (2.2) | 13.4 (1.6) | 0,093 |
| Hematocrit | 36.9 (6.4) | 40.0 (4.8) | 0,173 |
| Red Cell Distribution Width, (%) | 13.4 (1.1) | 13.1 (0.7) | 0,470 |
| Creatinine | 1.4 (1.5) | 0.9 (0.3) | 0,468 |
| C-Reactive Protein, mg/L | 154.4 (111.9) | 105.6 (83.0) | 0,237 |
| Lactate | 15.6 (7.1) | 12.9 (5.6) | 0,253 |
| D-dimer | 3.0 (2.8) | 1.8 (1.8) | 0,215 |
| Troponin | 168.4 (223.8) | 11.3 (9.3) | **0,040** |
| **Laboratory D3** | | | |
| Lymphocytes, cells/µl, Mean (SD) | 1,381.76 (922.29) | 1,568 (772) | 0,275 |
| Neutrophils, cells/µl | 8,012 (3,190) | 4,700 (2,052) | **0,001** |
| Monocytes, cells/µl | 796 (1,004) | 451 (182) | 0,978 |
| Neutrophil-Lymphocyte Ratio | 7.3 (3.9) | 4.2 (3.7) | **0,004** |
| Platelets, cells/µl | 256,461 (102,326) | 265,142.85 (88,955) | 0,783 |
| Hemoglobin, g/dL | 12.2 (2.1) | 12.6 (1.5) | 0,507 |
| Hematocrit, (%) | 37.2 (1.5) | 37.9 (4.6) | 0,731 |
| Red Cell Distribution Width, (%) | 13.5 (1.3) | 13.1 (0.8) | 0,384 |
| Creatinine, mg/dL | 2.1 (2.7) | 0.8 (0.3) | 0,456 |
| C-Reactive Protein, mg/L | 160.3 (116.8) | 66.0 (53.0) | **0,029** |
| **Laboratory D7** | | | |
| Lymphocytes, cells/µl, Mean (SD) | 952 (583) | 1,779 (631) | **0,007** |
| Neutrophils, cells/µl | 10,526 (4,159) | 4,711 (1,235) | **0,002** |
| Monocytes, cells/µl | 753 (425) | 577 (261) | 0,283 |
| Neutrophil-Lymphocyte Ratio | 16.7 (11.8) | 2.9 (1.2) | **0,001** |
| Platelets, cells/µl | 301,700 (117,169) | 404,300 (149,831) | 0,105 |
| Hemoglobin, g/dL | 11.2 (2.0) | 11.8 (1.2) | 0,455 |
| Hematocrit, (%) | 34.3 (5.8) | 35.7 (4.8) | 0,522 |
| Red Cell Distribution Width, (%) | 13.2 (1.2) | 13.1 (1.1) | 0,402 |
| Creatinine, mg/dL | 1.5 (0.9) | 0.8 (0.2) | 0,253 |
| C-Reactive Protein, mg/L | 114.2 (92.9) | 62.8 (56.5) | 0,257 |
| **Hospital Discharge** | | | |
| Lymphocytes, cells/µl, Mean (SD) | 2,093 (656) | 1,916 (748) | 0,555 |
| Neutrophils, cells/µl | 10,596 (7,182) | 4,938 (2,173) | **0,035** |
| Monocytes, cells/µl | 944 (796) | 578 (223) | **0,046** |
| Neutrophil-Lymphocyte Ratio | 5.7 (4.8) | 3.1 (2.2) | **0,042** |
| Platelets, cells/µl | 286,000 (126,059) | 300,937 (133,457) | 0,668 |
| Hemoglobin, g/dL | 10.8 (2.5) | 12.6 (1.2) | 0,055 |
| Hematocrit, (%) | 33.5 (7.8) | 37.9 (3.6) | 0,148 |
| Red Cell Distribution Width, (%) | 14.2 (1.8) | 12.9 (0.8) | **0,013** |
| Creatinine, mg/dL | 1.6 (1.0) | 0.8 (0.2) | **0,036** |
| C-Reactive Protein, mg/L | 48.9 (33.5) | 27.6 (23.9) | **0,049** |
|  |  |  |  |
| * Mann-Whitney, t-test or chi-square were applied to determine the P value when comparing groups | | |  |
| SD (Standard Deviation) |  |  |  |
| SpO2 (Oxygen Saturation) |  |  |  |
| SOFA score (Sequential Organ Failure Assessment Score) | |  |  |
