# Supplementary material for: The clinical course of hospitalized moderately ill COVID-19 patients is mirrored by routine hematologic tests and influenced by renal transplantation
Source: PLoS One. 2021 Nov 18;16(11):e0258987. doi: 10.1371/journal.pone.0258987 (PMC8601535; doi:10.1371/journal.pone.0258987)
Supplement: S3 Table — (DOCX) [file pone.0258987.s006.docx]

| **Supplementary table 3**. Demographic, clinical and laboratory results of KT recipients COVID-19 patients accordingly to the clinical course of the disease | | | |
| --- | --- | --- | --- |
|  | **Critical N=10** | **Non-critical (N =10 )** | **P-Value **** |
| **Demography** | | | |
| Male sex, n (ratio) | 4 (0.40) | 8 (0.80) | 0,068 |
| Age, Mean (SD) | 56.8 (0.1) | 52.38 (0.1) | 0,368 |
| **Admission data** | | | |
| Day of symptoms | 7.2 (2.8) | 8.8 (6.2) | 0,879 |
| Fever, n (ratio) | 7 (0.70) | 9 (0.90) | 0,264 |
| Cough | 6 (0.60) | 8 (0.80) | 0,329 |
| Shortness of breath | 8 (0.80) | 7 (0.70) | 0,606 |
| Diarrhea | 5 (0.50) | 2 (0.20) | 0,160 |
| Temperature, Mean (SD) | 36.4 (0.7) | 37.0 (0.9) | 0,650 |
| Cardiac Rate | 81.1 (10.8) | 89.3 (14.1) | **0,028** |
| Respiratory rate | 24.0 (3.7) | 23.3 (3.5) | 0,673 |
| SpO2 | 91.8 (3.2) | 94.3 (2.1) | 0,058 |
| Body mass index | 26.7 (3.0) | 24.6 (4.5) | 0,297 |
| SOFA score | 3.0 (2.1) | 2.4 (1.3) | 0,900 |
| **Comorbidities** | | | |
| Cardiac disease, n (ratio) | 0 (0.0) | 1 (0.10) | 0,305 |
| Chronic pulmonary disease | 0 (0.0) | 0 (0.0) | *NA* |
| Diabetes | 4 (0.40) | 3 (0.30) | 0,639 |
| Chronic kidney disease | 10 (1) | 10 (1) | *NA* |
| Hypertension | 7 (0.70) | 9 (0.90) | 0,264 |
| Obesity | 0 (0.0) | 1 (0.10) | 0,305 |
| Kidney transplant | 10 (1) | 10 (1) | *NA* |
| Stroke | 0 (0.0) | 1 (0.10) | 0,305 |
| Charlson Comorbidity index, Mean (SD) | 4.1 (1.5) | 3.4 (1.5) | 0,278 |
| Hospital days | 30.3 (16.7) | 9.7 (6.2) | **0,002** |
| **Laboratory Admission** | | | |
| Lymphocytes, cells/µl, Mean (SD) | 694 (497) | 737 (422) | 0,705 |
| Neutrophils, cells/µl | 5,468 (1,819) | 5,153 (2,963) | 0,778 |
| Monocytes, cells/µl | 338 (229) | 472 (388) | 0,545 |
| Neutrophil-Lymphocyte Ratio | 14.7 (16.8) | 8.68 (5.8) | 0,597 |
| Platelets, cells/µl | 181,400 (73,087) | 215,100 (54,085) | 0,256 |
| Hemoglobin, g/dL | 12.3 (1.1) | 12.2 (1.6) | 0,875 |
| Hematocrit (%) | 37.5 (3.2) | 37.5 (4.4) | 0,986 |
| Red Cell Distribution Width, (%) | 13.5 (1.1) | 13.9 (1.5) | 0,363 |
| Creatinine, mg/dL | 2.4 (1.6) | 1.8 (0.7) | 0,496 |
| C-Reactive Protein, mg/L | 111.2 (65.0) | 107.8 (50.5) | 0,902 |
| Lactate, mg/dL | 18.3 (10.5) | 10.3 (7.5) | 0,171 |
| D-dimer, µg/mL FEU | 1.1 (0.9) | 2.1 (2.7) | 0,286 |
| Troponin, ng/L | 23.3 (14.7) | 27.0 (9.2) | 0,667 |
| **Laboratory D3** | | | |
| Lymphocytes, cells/µl, Mean (SD) | 511 (278) | 492 (246) | 0,705 |
| Neutrophils, cells/µl | 7,289 (4721) | 5,464 (3,032) | 0,257 |
| Monocytes, cells/µl | 394 (239) | 295 (124) | 0,337 |
| Neutrophil-Lymphocyte Ratio | 22.1 (30.5) | 12.5 (8.2) | 0,597 |
| Platelets, cells/µl | 234,500 (95,974) | 226,857 (61,420) | 0,856 |
| Hemoglobin, g/dL | 12.0 (1.0) | 11.2 (1.7) | 0,634 |
| Hematocrit (%) | 36.7 (3.2) | 34.4 (4.9) | 0,263 |
| Red Cell Distribution Width, (%) | 13.6 (1.2) | 13.9 (1.5) | 0,873 |
| Creatinine, mg/dL | 2.4 (1.5) | 1.5 (0.4) | 0,286 |
| C-Reactive Protein, mg/L | 115.1 (118.4) | 54.4 (15.9) | 0,734 |
| **Laboratory D7** |  |  |  |
| Lymphocytes, cells/µl, Mean (SD) | 621 (276) | 780 (375) | 0,360 |
| Neutrophils, cells/µl | 8,666 (4,120) | 5,413 (2,500) | 0,109 |
| Monocytes, cells/µl | 460 (210) | 470 (261) | 0,932 |
| Neutrophil-Lymphocyte Ratio | 19.0 (15.8) | 8.6 (6.7) | 0,126 |
| Platelets, cells/µl | 247,888 (79,272) | 303,666.6 (25,796.6) | 0,077 |
| Hemoglobin, g/dL | 11.4 (1.6) | 11.2 (2.0) | 0,813 |
| Hematocrit (%) | 35.2 (5.5) | 34.3 (5.6) | 0,764 |
| Red Cell Distribution Width, (%) | 13.5 (1.6) | 13.8 (1.4) | 0,555 |
| Creatinine, mg/dL | 2.7 (1.3) | 1.4 (0.3) | **0,010** |
| C-Reactive Protein, mg/L | 135.1 (150.2) | 39.1 (29.0) | 0,200 |
| **Hospital Discharge** | | | |
| Lymphocytes, cells/µl, Mean (SD) | 1,260 (784) | 972 (489) | 0,350 |
| Neutrophils, cells/µl | 13,793 (4,905) | 4,618 (2361) | **0,0003** |
| Monocytes, cells/µl | 965 (679) | 604 (213) | 0,160 |
| Neutrophil-Lymphocyte Ratio | 21.0 (26.9) | 6.0 (5.6) | **0,010** |
| Platelets, cells/µl | 179,666 (168,300) | 305,500 (81,841) | 0,070 |
| Hemoglobin, g/dL | 8.5 (2.01) | 11.8 (1.3) | **0,001** |
| Hematocrit (%) | 26.0 (5.8) | 36.5 (3.9) | **0,0002** |
| Red Cell Distribution Width, (%) | 14.4 (1.4) | 14.0 (2.0) | 0,347 |
| Creatinine, mg/dL | 2.8 (1.2) | 1.4 (0.2) | **0,042** |
| C-Reactive Protein, mg/L | 88.0 (67.0) | 24.8 (17.6) | 0,041 |
|  |  |  |  |
| * Mann-Whitney, t-test or chi-square were applied to determine the P value when comparing groups | | |  |
| SD (Standard Deviation) |  |  |  |
| SpO2 (Oxygen Saturation) |  |  |  |
| SOFA score (Sequential Organ Failure Assessment Score) | |  |  |
