# Supplementary material for: The clinical course of hospitalized moderately ill COVID-19 patients is mirrored by routine hematologic tests and influenced by renal transplantation
Source: PLoS One. 2021 Nov 18;16(11):e0258987. doi: 10.1371/journal.pone.0258987 (PMC8601535; doi:10.1371/journal.pone.0258987)
Supplement: Attachment — Submitted filename: Response to Reviewers.docx [file pone.0258987.s008.docx]

Chiara Lazzeri

Academic Editor

PLOS ONE

We thank the referees’ comments, which are responded point by point below.

1. Is the manuscript technically sound, and do the data support the conclusions?

The manuscript must describe a technically sound piece of scientific research with data that supports the conclusions. Experiments must have been conducted rigorously, with appropriate controls, replication, and sample sizes. The conclusions must be drawn appropriately based on the data presented.

Reviewer #1: Partly

Response: we have reviewed the manuscript and hope to have improved its quality and presentation.

Reviewer #2: Yes

2. Has the statistical analysis been performed appropriately and rigorously?

Reviewer #1: N/A

Reviewer #2: Yes

3. Have the authors made all data underlying the findings in their manuscript fully available?

The PLOS Data policy requires authors to make all data underlying the findings described in their manuscript fully available without restriction, with rare exception (please refer to the Data Availability Statement in the manuscript PDF file). The data should be provided as part of the manuscript or its supporting information, or deposited to a public repository. For example, in addition to summary statistics, the data points behind means, medians and variance measures should be available. If there are restrictions on publicly sharing data—e.g. participant privacy or use of data from a third party—those must be specified.

Reviewer #1: Yes

Reviewer #2: Yes

4. Is the manuscript presented in an intelligible fashion and written in standard English?

Reviewer #1: Yes

Reviewer #2: No

Response: we have reviewed the manuscript and hope to have improved its quality and presentation.

**5. Review Comments to the Author**

**Reviewer #1: The authors made a great effort to support their thesis. They have great credit for following their population prospectively. However, the blood chemistry parameters they consider are also the first to change over the course of any infection and inflammation. There is no doubt that these changes are part of the course of COVID disease, but their ability to predict outcome is rather weak, correlation with the specific pathology under consideration should be improved.**

I suggest to the authors to improve the study design, not limiting themselves only to describing but trying to correlate what was observed to the specific pathology in a stronger and more unambiguous way. I also suggest that we organize the discussion better to make it more linear and less confusing. Better to stick to the description of the results and their interpretation more concisely.

Response: Thank you for your comments.

We have completely revised the manuscript, focusing on the cohort description and the main results obtained. To make the message clearer, we modified the tables that were in the main text and those presented as supplementary.

For some comparisons (as in supplementary table 1) we changed the statistical approach to compare between patients deteriorating to critical illness and those with clinical recovery, and among the first ones between those surviving and not surviving. Furthermore, we added a Principal Components Analysis (PCA) in order to identify the set of variables that most correlates with outcomes.

Results are presented focusing on the routine hematologic changes and clinical outcomes; the issue of kidney transplant recipients (KTR) COVID-19 patients is presented more concisely.

The discussion was reorganized, as suggested. It is more concise (original version – 2167 words; revised version – 1489 words) and focused on the results.

The quality of figures is also improved.

The file “text marked with tracks” highlight part of these changes. Part of the texts that were removed could not be marked.

We hope to have improved the quality of the manuscript and attended your expectations.

**Reviewer #2: Could benefit from minor sytlistic/language revision for clarity sake. The findings of persistent elevation in NLR correlating with mortality is not unsurprising and did not change management, but the data may still be valuable for risk profiling.**

We thank you for the comments. Please, see the above response to the reviewer 1.

The text was reviewed, it is more focused, concise and, hopefully, also clear.

We hope to have improved the quality of the manuscript and attended your expectations.
